# Pacing Profiles in Competitive Track Races: Regulation of Exercise Intensity Is Related to Cognitive Ability

**DOI:** 10.3389/fphys.2016.00624

**Published:** 2016-12-20

**Authors:** Debbie Van Biesen, Florentina J. Hettinga, Katina McCulloch, Yves Vanlandewijck

**Affiliations:** ^1^Research Unit Adapted Physical Activity and Psychomotor Rehabilitation, Department of Rehabilitation Sciences, Faculty of Kinesiology and Rehabilitation Sciences, KU LeuvenLeuven, Belgium; ^2^Centre for Sports and Exercise Sciences, School of Biological Sciences, University of EssexColchester, UK

**Keywords:** running, 400 m, 1500 m, track and field, intelligence

## Abstract

Pacing has been defined as the goal-directed regulation of exercise intensity over an exercise bout, in which athletes need to decide how and when to invest their energy. The purpose of this study was to explore if the regulation of exercise intensity during competitive track races is different between runners with and without intellectual impairment, which is characterized by significant limitations in intellectual functioning (IQ ≤ 75) and adaptive behavioral deficits, diagnosed before the age of 18. The samples included elite runners with intellectual impairment (*N* = 36) and a comparison group of world class runners without impairment (*N* = 39), of which 47 were 400 m runners (all male) and 28 were 1500 m-runners (15 male and 13 female). Pacing was analyzed by means of 100 m split times (for 400 m races) and 200 m split times (for 1500 m races). Based on the split times, the average velocity was calculated for four segments of the races. Velocity fluctuations were defined as the differences in velocity between consecutive race segments. A mixed model ANOVA revealed significant differences in pacing profiles between runners with and without intellectual impairment (*p* < 0.05). Maximal velocity of elite 400 m runners with intellectual impairment in the first race segment (7.9 ± 0.3 m/s) was well below the top-velocity reached by world level 400 m runners without intellectual impairment (8.9 ± 0.2 m/s), and their overall pace was slower (*F* = 120.7, *p* < 0.05). In addition, both groups followed a different pacing profile and inter-individual differences in pacing profiles were larger, with differences most pronounced for 1500 m races. Whereas, male 1500 m-runners without intellectual impairment reached a high velocity in the first 100 m (7.2 ± 0.1 m/s), slowly decelerated in the second race segment (−0.6 ± 0.1 m/s), and finished with an end sprint (+0.9 ± 0.1 m/s); the 1500 m runners with intellectual impairment started slower (6.1 ± 0.3 m/s), accelerated in the second segment (+0.2 ± 0.7 m/s), and then slowly decreased until the finish (*F* = 6.8, *p* < 0.05). Our findings support the hypothesis that runners with intellectual impairment have difficulties to efficiently self-regulate their exercise intensity. Their limited cognitive resources may constrain the successful integration of appropriate pacing strategies during competitive races.

## Introduction

A vital component for success in running events is the pacing strategy (Abbiss and Laursen, 2008; Tucker, 2009). The optimal pacing strategy can be a learned pattern, based on extensive experience gained during training and previous competitions (Foster et al., 2009, 2014); however, many factors can affect the pacing strategies adopted during running events. An individuals' pacing strategy is dependent on performance goals (e.g., world record attempt vs. qualification during heats; Thompson, 2015), environmental conditions (e.g., temperature) (Tucker, 2009; Roelands et al., 2013) and the presence of opponents (Konings et al., 2016a,b). In competition, athletes must set and adjust their pace based on feelings such as perceived exertion (Abbiss and Laursen, 2008) or pain (Mauger, 2014). Hence, the actual pacing profile observed during competition does not always resemble the pre-planned strategy adopted by the athlete and/or the coach. Competitors need to take into account the distance remaining until finish and also the actions of their opponents (St Clair Gibson et al., 2006; De Koning et al., 2011; Swart et al., 2012). When considering an athletic event involving direct competition between two or more individual athletes, the environment becomes even more complex (Renfree et al., 2014; Konings et al., 2016a,b).

Several recent reviews have described pacing as a process of decision-making (Renfree et al., 2014; Smits et al., 2014). It was recently proposed that effective cognitive control during performance requires both proactive, goal-driven processes and reactive, stimulus-driven processes (Brick et al., 2016). Although the importance of decision-making upon effort regulation was acknowledged (De Koning et al., 2011; Renfree and St Clair Gibson, 2013), very little is understood about decision-making processes involved in pacing or the underlying psychological mechanisms. To understand how exercisers regulate their exercise capacity, and to identify the role cognition plays in optimal self-regulation, the study of pacing in athletes with intellectual impairments could be an interesting design. Although pacing is commonly accepted as an important cognitive determinant in running (St Clair Gibson et al., 2006; Tucker et al., 2006; Abbiss and Laursen, 2008; Hanon et al., 2008; Tucker, 2009; De Koning et al., 2011; Hanon and Thomas, 2011; Saraslanidis et al., 2011; Thiel et al., 2012; Reardon, 2013; Renfree et al., 2014; Smits et al., 2014) only one study has investigated pacing in individuals with intellectual impairment. Micklewright et al. (2012) demonstrated an explicit link between pacing and cognitive development by looking into pacing behavior of school children in different stages of cognitive development. The study confirmed that developing a pacing strategy is at least in part determined by cognitive mechanisms. In their study, after doing a control test for age (5–14 years), pacing differences were distinguished between groups of school children in different stages of cognitive development. In another study it was demonstrated in a large sample of elite swimmers, athletes, basketball- and table tennis players with intellectual impairment that their cognitive abilities relevant to sport in general (e.g., visual processing, reaction and decision making speed, short-term memory and fluid reasoning) were significantly reduced compared to equally well-trained athletes without impairment (Van Biesen et al., 2016b), so it can be assumed that specific cognitive abilities relevant to pacing and performance in running (i.e., decision making, anticipation) will also be influenced by having an intellectual impairment. A first study exploring this analyzed the ability of runners with an intellectual impairment to maintain a pre-planned velocity over 400 m, an essential aspect of pacing (Van Biesen et al., 2016a). It was demonstrated that runners with an intellectual impairment were not able to maintain the required sub-maximal velocity and accelerated toward the end, in contrast to athletes without impairment of similar training volume. This provided the first evidence for the impact of cognitive ability on pacing ability. The present study will now focus on exploring data of athletes in actual competitions to explore how cognitive ability impacts on pacing and performance in competition.

The purpose of the present study was to explore if the regulation of exercise intensity during competitive 400 and 1500 m track races is different when pacing profiles are compared between high level runners with and without intellectual impairment. It is hypothesized that runners with intellectual impairment will have a different, more variable pacing strategy compared to runners without intellectual impairment. If we detect an effect of having an intellectual impairment on pacing profiles during the race, this will provide evidence to support the assumption that the regulation of runners' exercise intensity over the race is, at least partly, dependent on their cognitive skill level. In addition, a difference in pacing profiles between the groups will create an evidence-based rationale for organizing separate competitions for runners with intellectual impairment in the Paralympic Games.

## Materials and methods

### Participants

Data for this study were derived from a sample of 47,400 and 281,500 m runners, of which 36 elite runners with mild intellectual impairment (28 males and 8 females) and a comparison group of 39 runners without impairment (34 males and 5 females). The runners with intellectual impairment competed at the 2014 Open European Championship Athletics, in Bergen Op Zoom, The Netherlands, organized by the International Federation for Para-Athletes with Intellectual Impairment (INAS). They competed in 400 or 1500 m races and all met the criteria for diagnosis of intellectual disability as set by the American Association on Intellectual and Developmental Disabilities: IQ ≤ 75, significant deficits in adapted behavior and manifested before the age of 18. More specifically, the IQ scores of the runners with intellectual impairment were 64.7 ± 8.7 (male 400 m), 59.6 ± 8.7 (male 1500 m) and 60.4 ± 7.9 (female 1500 m). None of the participants had severe intellectual impairment or a genetic syndrome (e.g., Down Syndrome). The runners with intellectual impairment (aged 24.4 ± 4.5 years) had on average 9.6 ± 4.8 years of experience and 9.4 ± 4.0 h/week training volume. The control data was obtained from the International Association of Athletics Federation's (IAAF) 12th World Championships in Berlin in 2009 (Helmar et al., 2009a,b). For the 1500-m world record performances of men and women, split times were obtained from http://www.iaaf.org/ and http://wn.com/ respectively. Descriptive information of the participants in the control group (age, training volume, IQ scores) was not available. The study was approved by the local ethics committee (Commissie Medische Ethiek, KU Leuven).

### Procedure

**Figure 1:**
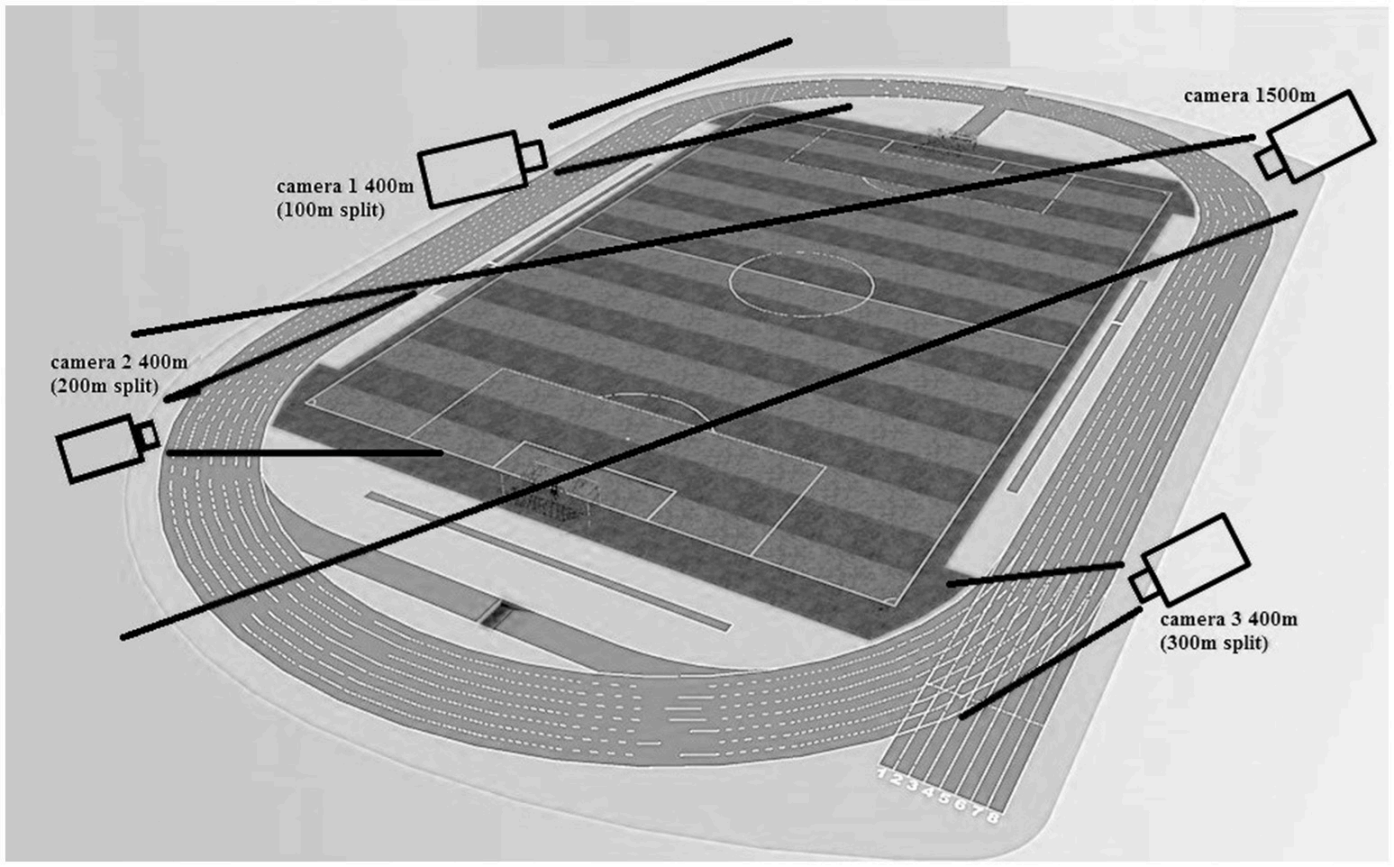
**Camera positions for split time calculations during 400 and 1500-m races**.

Pacing profiles were analyzed by means of 100 m split times (for 400 m races) and 200 m split times (for 1500 m races). The most recent World Record data were retrieved from the IAAF website (Reardon, 2013; International Association of Athletics Federations, 2016). Split times were publically available on the IAAF website for the control group, and split times were calculated for the runners with intellectual impairment on the basis of video images recorded during the race. Their races were filmed with three 25 Hz SONY Cameras for the 400-m race, and one camera for the 1500-m race. The positions of the cameras are depicted in Figure 1. During the 1500-m race a large cone was placed in view of the camera as a reference point for the calculation of the 100, 500, 900, and 1300-m split time. Before the start of every 400-m race, the camera captured the first athlete in starting position (lane 1 or the most inner athlete). From the moment the athletes took off, the camera was switched to the designated split time mark to capture every athlete passing by.

### Data reduction and calculation

Based on the split times and distance, the average velocity was calculated for four segments of the race: 0–100 m, 100–200 m, 200–300 m, and 300–400 m for the 400 m races and 0–100 m, 100–500 m, 500–1000 m, and 1000–1500 m for the 1500 m races. Velocity fluctuated within the segments indicating accelerations (i.e., positive fluctuations) or decelerations (i.e., negative fluctuations).

### Data analyses

Statistics were performed using SPSS (version 19.0, SPSS Inc., Chicago Ill, USA) with level of significance set at *p* < 0.05. For the 400 m race, a mixed model ANOVA was performed to analyze the differences in running patterns over different time points during the race (within factor), between male runners with and without intellectual impairment (between factor), for heats and finals. The mixed model ANOVA was also performed to analyze the differences in running patterns over different time points (within) between runners with and without intellectual impairment (between) in the 1500 m finals. Intra-individual coefficients of variation of running speed within each race were calculated based on 100-m split times (for the 400 m races) and 200-m split times (for the 1500-m races).

## Results

### 400 m group differences in race strategy

**Figure 2:**
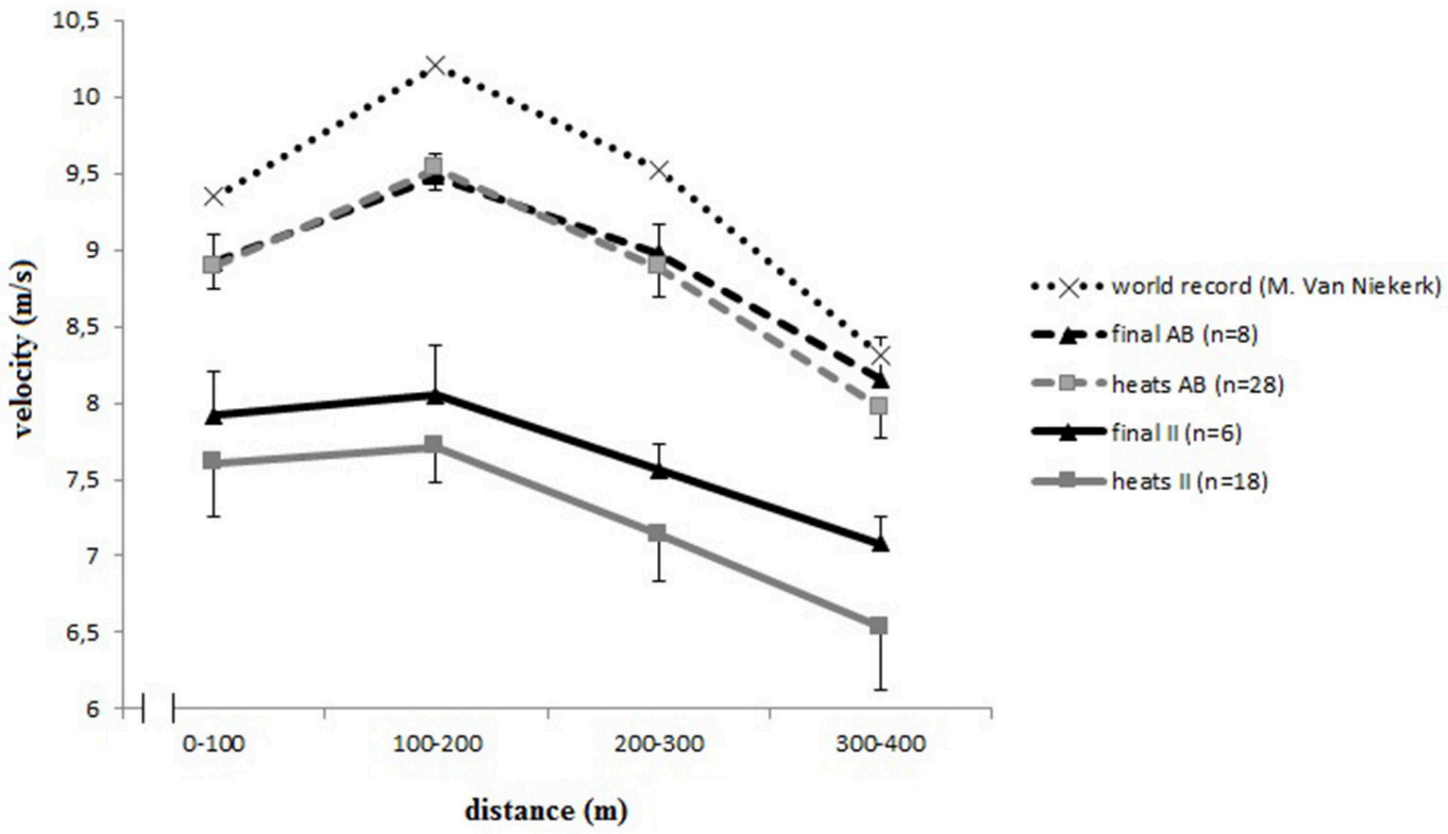
**Men's 400-m pacing profiles**. INAS, International Federation for para-athletes with intellectual impairment; II, intellectual impairment; AB, able bodied.

Figure 2 shows the overall pacing strategy during the men's 400-m races. Average velocity plots per segment are shown for the heats and finals. No significant differences in velocity were found between finals and heats for runners without intellectual impairment, whereas average velocity at all time-points was higher in the final race than during heats for runners with intellectual impairment. Both groups initially performed an acceleration followed by a deceleration, however, the pacing strategy significantly differed between both groups of runners in heats and finals as shown by the significant interaction effect (Table 1). The runners without intellectual impairment gradually decelerated halfway after a fast start. The deceleration, traveling between 9.5 and 8 m/s, concluded with a steeper decline in the latter part. For the runners with intellectual impairment, the decline occurred with a steep descent from 8 until 7 m/s. The result of the *post hoc* analyses as shown in Table 2 indicated that fluctuations in the final race segment were significantly different between both groups of runners in the heats (*F* = 7.1, *p* < 0.05); however, not for the finals (*F* = 7.1, *p* = 0.1).

**Table 1:** **Mixed model Anova results for velocity fluctuations in four races: 400 m male final and heats, 1500 m male and female final between runners with and without intellectual impairment**.

	***df***	***F***	**η^2^**	***p***
**400 M FINAL MEN**				
ME_w_ velocity	1, 14	67.23	0.95	<0.001^*^
ME_b_ impairment	1, 14	241.56	0.95	<0.001^*^
IE velocity × impairment	1, 14	12.50	0.79	0.001^*^
**400 M HEATS MEN**				
ME_w_ velocity	1, 46	333.74	0.96	<0.001^*^
ME_b_ impairment	1, 46	1265.90	0.97	<0.001^*^
IE velocity × impairment	1, 46	123.33	0.63	<0.001^*^
**1500 M FINAL MEN**				
ME_w_ velocity	1, 14	5.25	0.61	0.02^*^
ME_b_ impairment	1, 14	45.21	0.79	<0.001^*^
IE Velocity × impairment	1, 14	35.36	0.92	<0.001^*^
**1500 M FINAL WOMEN**				
ME_w_ velocity	1, 12	10.31	0.79	0.004^*^
ME_b_ impairment	1, 12	58.94	0.86	<0.001^*^
IE velocity × impairment	1, 12	66.79	0.96	<0.001^*^

Df, degrees of freedom;

* *p < 0.05, ME_w_, main effect of the within-subjects factor; ME_b_, main effect of the between subjects factor; IE, interaction effect*.

**Table 2:** **Comparison of velocity fluctuations over four segments of the races between runners with and without intellectual impairment**.

	**With intellectual impairment**			**Without intellectual impairment**			***F***	**ES Cohen d**
	**Mean (m/s)**	**SD**	**95% CI**	**Mean (m/s)**	**SD**	**95% CI**		
**400 M FINAL (MEN,** ***N*** = **14)**								
Q1	7.9	0.3	[7.6, 8.2]	8.9	0.2	[8.7, 9.1]	120.7^*^	3.9
Q2	0.1	0.2	[−0.1, 0.4]	0.6	0.1	[0.5, 0.7]	21.4^*^	3.2
Q3	−**0.5**	**0.2**	[−0.7, −0.3]	−**0.5**	**0.2**	[−0.7, −0.3]	1.2	0
Q4	−**0.5**	**0.3**	[−0.8, −0.2]	−**0.8**	**0.2**	[−1.0, −0.7]	7.1	1.2
**400 M HEATS (MEN,** ***N*** = **46)**								
Q1	7.6	0.4	[7.4, 7.8]	8.9	0.2	[8.8, 8.9]	120.7^*^	4.1
Q2	0.1	0.3	[−0.1, 0.3]	0.6	0.2	[0.6, 0.7]	21.4^*^	2.0
Q3	−**0.6**	**0.2**	[−0.7, −0.5]	−**0.6**	**0.3**	[−0.7, −0.5]	1.2	0
Q4	−**0.6**	**0.2**	[−0.7, −0.5]	−**0.9**	**0.3**	[−1.0, −0.7]	7.1^*^	1.2
**1500 M FINAL (MEN,** ***N*** = **14)**								
Q1	6.1	0.3	[5.9, 6.3]	7.2	0.1	[7.1, 7.3]	−6.8^*^	5.0
Q2	0.2	0.7	[−0.3, 0.6]	−**0.6**	**0.1**	[−0.7, −0.6]	3.8^*^	1.6
Q3	−**0.4**	**0.3**	[−0.6, −0.2]	0.3	0.0	[0.3, 0.3]	−7.6^*^	3.3
Q4	−**0.1**	**0.4**	[−0.3, 0.2]	0.9	0.1	[0.9, 1.0]	−7.6^*^	3.4
**1500 M FINAL (WOMEN,** ***N*** = **12)**								
Q1	4.9	0.1	[4.8, 5.0]	6.5	0.1	[6.3, 6.6]	−28.8^*^	16.0
Q2	0.3	0.2	[0.1, 0.5]	−**0.8**	**0.1**	[−1.0, −0.6]	10.6^*^	7.0
Q3	−**0.5**	**0.2**	[−0.6, −0.3]	0.6	0.0	[0.6, 0.7]	−15.8^*^	7.8
Q4	0.3	0.6	[−0.1, 0.8]	0.2	0.0	[0.1, 0.3]	0.7	0.2

Q1, first race segment (0–100 m); Q2, second race segment (100–200 m or 100–500 m); Q3, third race segment (200–300 m or 500–1000 m); Q4, fourth race segment (300–400 m or 1000–1500 m); CI, Confidence interval; SD, standard deviation;

* *p < 0.05, negative velocity fluctuations (= deceleration) is highlighted in bold*.

Overall, runners with intellectual impairment demonstrated a slower running speed than runners without intellectual impairment. The ANOVA showed a significant main effect of the within factor velocity in the 400 m heats and 400 m final races (Table 1). In the first race segment (0–100 m) of the final, runners with intellectual impairment accelerated to a velocity of 7.9 m/s, whereas runners without intellectual impairment accelerated to 8.9 m/s (*F* = 120.7, *p* < 0.05, Table 2). Another difference between both groups was observed in the second race segment (100–200 m). In both the final and the heats, runners with intellectual impairment accelerated (0.1 ± 0.2 m/s); however, this acceleration was less pronounced than demonstrated by the runners without intellectual impairment (0.6 ± 0.1 m/s); The latter group reached their maximal speed after 200 m (*F* = 21.4, *p* < 0.05).

### 400 m individual differences in race strategy

Coefficients of variance (CV) were calculated as a measure of intra-individual variance. The average CV of the male runners with intellectual impairment who ran the 400 m final, semi-finals, and/or qualifications in Bergen op Zoom was 8.1 ± 2.9% whereas the coefficient of variation during the World Championships in Berlin was 6.9 ± 1.6%.

### 1500 m race group differences

**Figure 3:**
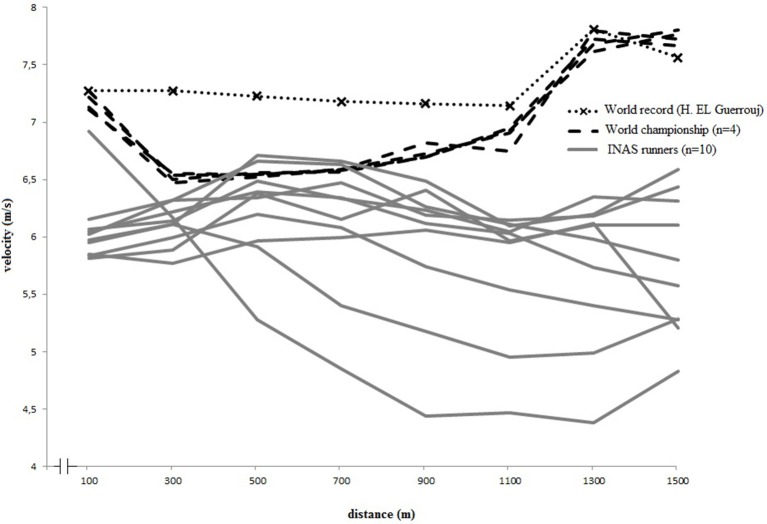
**Individual pacing strategies of elite men's 1500 m finalists (II and non-II) vs. World Record**. INAS, International Federation for para-athletes with intellectual impairment.

**Figure 4:**
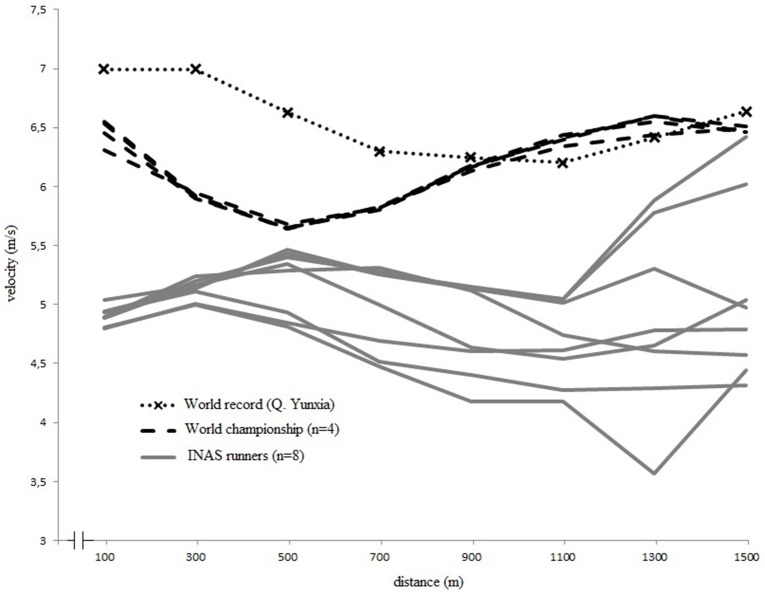
**Individual pacing strategies of elite women's 1500 m finalists (II and non-II) vs. World Record**. INAS, International Federation for para-athletes with intellectual impairment.

Figures 3, 4 display the pacing strategies applied by respectively male and female runners during their 1500 m final race. The velocity fluctuations within every race segment are quantified in Table 2. An overall comparison of the distance by velocity plots (Figure 3) shows that male runners with and without intellectual impairment followed a different, almost inverse, pacing profile, confirmed by a significant interaction effect (Table 1). After reaching a relatively high velocity in the first 100 m (6.1 m/s), male runners without intellectual impairment controlled their pace and slowly decelerated in the second segment of the race (100–500 m) to finish with an end sprint (1000–1500 m), whereas runners with intellectual impairment started slower, accelerated in the second segment, and then slowly decreased velocity until the end (*F* = 6.8, *p* < 0.05). The comparison between female 1500 m runners with and without intellectual impairment (Figure 4) also revealed inverse pacing profiles between both groups of runners, with runners with intellectual impairment accelerating until 500 m, followed by a deceleration until 1100 m, and a variable strategy until finish. The runners without intellectual impairment did the opposite, decelerating between 100 and 500 m, followed by accelerating until 1300 m, and then maintaining their velocity until finish. Significant differences were found (Table 1) between the groups in the first three segments of the race (0–1000 m). Only in the final segment (1000–1500 m) both female groups slightly accelerated.

### 1500 m race intra- and inter-individual differences

In Figures 3, 4, the individual race velocity profiles during the final 1500 m races are plotted. Based on visual inspection, it can be seen that the inter-individual differences were large in the group of runners with intellectual impairment compared to the runners without intellectual impairment. The inter-individual differences were also more pronounced for runners with intellectual impairment. CV was calculated to express the intra-individual differences in velocity over the race. However, during the World Championship final male 1500 m runners without intellectual impairment demonstrated a CV of 7.3 ± 0.5%, and runners with intellectual impairment had an average CV of 5.5 ± 3.1%. Female world championships finalists had a similar CV (6.5 ± 2.7% for runners with intellectual impairment compared to 5.8 ± 0.5% for runners without).

## Discussion

The purpose of this study was to explore the differences in pacing strategy between well-trained middle distance runners with and without intellectual impairment. Clear differences in pacing profiles were observed between runners with and without intellectual impairment. Results indicated that runners with intellectual impairment paced their race differently and with greater variance than runners without intellectual impairment. The differences were observed in 400 and 1500 m races, and for both distances, the differences were most pronounced in the first half of the race. Our findings largely support the hypothesis that having an intellectual impairment impacts on the ability of runners with intellectual impairment to effectively regulate their exercise intensity over the race, supporting the assumption that this ability is at least partly dependent on cognitive skill level. To our knowledge, this was the first study to compare pacing profiles during competitive races of well-trained high level runners with and without intellectual impairment.

Within the literature, pacing has been described as an important cognitive factor in middle-distance and endurance performance that is regulated by the brain (St Clair Gibson et al., 2006; Tucker, 2009) and has been defined as the goal-directed regulation of exercise intensity over an exercise bout, in which athletes need to decide how and when to invest their energy (Smits et al., 2014). The optimal pacing strategies for different running distances were described extensively in the literature (Tucker et al., 2006; Abbiss and Laursen, 2008; Hanon et al., 2008; Thiel et al., 2012; Reardon, 2013; Thompson, 2015). Thompson (2015) described that for the 400-m event, a positive pacing profile is the most optimal strategy; where the speed of the athlete gradually decreases during the race. Other studies also suggested a positive pacing profile as the optimal strategy during a 400-m event (Tucker et al., 2006; Abbiss and Laursen, 2008; Reardon, 2013). Runners are decelerating toward the latter segment of the 400-m race, primarily due to developing fatigue (Thompson, 2015). All world records for 400-m races have been run with a positive pacing strategy (Reardon, 2013), with the results of this study showing that runners with intellectual impairment overall also use a positive pacing strategy over the 400 m running event. Their typical profile of decline of velocity in the two different segments of the second half of the race (slow decline/fast decline) was also be observed in the world record race run by Wayde Van Niekerk in the Olympic Final in Rio 2016 (Vazel, 2016).

Regarding the 1500-m event, an optimal pacing strategy for a 1500 m race is even paced in the middle section; however, overall it is more parabolic according to literature (Hanon et al., 2008; Thompson, 2015). Thomas et al. (2013) showed that though even pacing might theoretically be optimal for endurance performance (De Koning et al., 1999), but in athlete's reality a parabolic shaped pattern might be more appropriate since the cyclists in their study were not able to finish the race when forced into an even paced pattern. In addition, it is important to note that these findings are from cyclists, as differences in optimal pacing might exist between different sports due to their specific characteristics (Stoter et al., 2016). The male world record by El Guerrouj however followed the even paced strategy, rather than the parabolic strategy, with an acceleration at the end (http://www.iaaf.org/); whereas the female world record by Yunxia followed a parabolic pacing strategy (http://wn.com/), at overall higher velocities. In our study, the runners with intellectual impairment adopted different pacing strategies compared to what is considered optimal in literature, or what is logically assumed optimal (i.e., world record performance). The male runners with intellectual impairment were not able to perform an end sprint; which is, probably because they started at very high velocities. Instead of choosing for a controlled, slower pace during the middle part of the race, we assume that the runners might have been physiologically forced to slow downmaking sure not to deplete energy stores prematurely to the races completion (St Clair Gibson et al., 2006). The female runners with intellectual impairment sustained their high start velocity over a long period during the initial segment of the race, before decelerating in the mid-section. They were then able to perform an end sprint at the end of the race; although their average speed overall was lower compared to runners without intellectual impairment.

With respect to the individual patterns of runners with intellectual impairment, high inter-individual variation during the race was observed, with different competitors within the same race applying different race strategies. Runners with intellectual impairment also showed more variance in velocity fluctuations during the race compared to the runners without intellectual impairment. The more consistent strategy applied by runners without intellectual impairment corresponded with Foster et al. (2014) who found a CV of 1.5–3.0% in 1-mile world record performances. In another study by Thiel et al. (2012) the CV during Olympic finals ranged between 3.6 and 11.4%; and, in the finals of the long distance races, the pace varied every 100 m between 1.6 and 2.7% (Thiel et al., 2012). In our study, the variation in running speed is large in runners with intellectual impairment, especially when comparing it to the world records. Using field data, the present study demonstrated that runners with intellectual impairment race with a larger intra-individual variability. Speed fluctuations result in relatively larger air frictional losses (Van Ingen Schenau et al., 1992); leading to a decrease in running economy and a subsequent decrease in performance (Foster et al., 2014). Large velocity fluctuations of competitors during the races can be related to their inability to control their own pace and to maintain a preplanned velocity, as we have demonstrated in a previous study (Van Biesen et al., 2016a). It can also be the result of athletes running a very tactical race (Reardon, 2013), athletes trying to separate themselves from the rest of the athletes when running in a pack (Foster et al., 2014), or due to specific uncommon events (e.g., the fall of one or more competitors). The inter-individual variability observed in runners with intellectual impairment corresponds with findings in many other studies (not only in running) involving participants with intellectual impairment. It was previously observed that intellectual impairment is related to larger inter-individual variation in reaction times (Carmeli et al., 2008), physical fitness (Graham and Reid, 2000; Lahtinen et al., 2007), and performance on sport-specific tasks such as table tennis technical proficiency (Van Biesen et al., 2012).

Comparing to what is known from literature and assuming that the world record pacing patterns are close to optimal, the results of this study indicated that runners with intellectual impairment adopt non-optimal pacing patterns during their races. This finding could be explained by numerous external factors which have an influence on the “optimal” distribution of work, such as other competitors (Konings et al., 2016a,b). Konings et al. (2016b) were the first to show that not only the presence, but also the behavior of an opponent might affect decisions regarding the regulation of exercise intensity in laboratory-controlled conditions. As one crucial element in the diagnosis of intellectual disability is a deficit in adaptive behavior (American Assocation on Intellectual Developmental Disabilities, 2011), the behavior of opponents during races for runners with intellectual impairment can be even more unpredictable compared to typical high level races. Also, less accomplished runners can feel forced to stay with the leading group at a pace markedly faster than their best performance. This increases the risk of premature excessive fatigue that could result in a decisive drop out later in the race (Thompson, 2015). An example of this was observed in the 1500 m final race for male runners with intellectual impairment, in which one runner started the race at a very high velocity, but he was not able to maintain this velocity and ended up finishing last (see Figure 3). This behavior is in line with our preceding study, in which athletes with an intellectual impairment in general were not able to maintain a pre-set sub-maximal velocity (Van Biesen et al., 2016a), but accelerated toward the finish line. It is possible that the behavior of this runner has influenced the profiles of the other finalists, who might have adapted their own pacing to this occurrence, as has been demonstrated to occur in well-trained cyclists (Konings et al., 2016a). In sports where athletes compete in heats, in direct competition with their opponents, this is known to influence their pacing as for example has been demonstrated in 500, 1000, and 1500 m short-track skating competitions (Konings et al., 2016b; Noorbergen et al., 2016). Not much is known yet on how intellectual impaired athletes respond to their opponents, but as athlete- environment interactions are crucial in pacing (Smits et al., 2014) we expect this is an important aspect and future research is needed. Motivational factors are also known to affect optimal pacing (Mauger, 2014). It is known that the increases in motivation and prior experience will reduce the subjective experience of exercise-induced pain during the race and/or increase the willingness of the runner to endure it (Mauger, 2014). Reduced levels of intrinsic motivation are often addressed in research involving participants with intellectual impairment (Hutzler and Korsensky, 2010), however the sample of participants in this specific project involved elite athletes and they were observed during competition at the European Championships, which is a context in which we can assume they perform maximally. Perhaps a more applicable explanation could be that cognitive control and adequate focus of attention are important metacognitive skills to successful pacing (Brick et al., 2016). These metacognitive skills, and most specifically the proactive cognitive control (i.e., anticipatory, goal-oriented processing of information or planning) place a great demand on cognitive resources (Braver, 2012) and these higher order cognitive skills were previously demonstrated to be reduced in elite athletes with intellectual impairment (Van Biesen et al., 2016b), who already have, by the nature of their impairment, limited cognitive resources (Van Biesen et al., unpublished manuscript). People with intellectual impairment are also known to have deficits in a range of other complex higher-order skills that are relevant to pacing (e.g., problem-solving, logical reasoning, and language-dependent strategies such as self-talk; Aitchison et al., 2013).

An interesting finding of the present study is that differences in pacing profiles during the 400 m races were rather small between both group of runners, particularly when compared to differences in the 1500 m. An explanation may be that runners with intellectual impairment, despite their lower levels of cognitive function (i.e., lower IQ), do have the relevant skills to adequately perform a 400 m race, in which an all-out approach is required. These findings correspond with the recent findings by Van Biesen et al. (2016a) that runners with intellectual impairment seem to have difficulties to self-regulate their pace when they are asked to maintain a submaximal velocity, which is required for a 1500 m. They had the tendency to accelerate, and found it difficult to control their velocity. The overall IQ scores of 400 m runners (64.72 ± 8.71) where somewhat higher than for 1500 m runners (59.94 ± 8.12) but this difference was not statistically significant (*p* = 0.09).

Overall, velocity of the runners with intellectual impairment is significantly lower compared to the runners without intellectual impairment, even though both groups consisted of elite athletes. The race observations of the runners with intellectual impairment took place at a European Championship, whereas the split times of the runners without intellectual impairment were obtained from a World Championship. The level at a World Championship is higher than that on a European Championship; however, the large difference in velocity between the two groups is probably not caused by the effect of the cognitive impairment on pacing only. Other aspects may also contribute, for instance the smaller population (i.e., easier to become a top II-runner), reduced maximal voluntary muscle contraction (Borji et al., 2014), the lack of motivation to perform maximally (Rimmer, 1994), reduced leg strength (Fernhall and Pitetti, 2001) or chronotropic incompetence (Dipla et al., 2013). However, the most important aspect to consider is the training volume. The comparison sample in this study was selected on the basis of comparable competition level (the highest obtainable). Training volume data were not available but we can assume that it is higher than the 10 h per week reported by the runners with intellectual impairment. Overall, the level of professionalism in sport for elite athletes with intellectual impairment compared to regular elite sport is not equal. Differences exist in training quality, access to top-coaches, prize money and sponsorship among other factors (Van Biesen et al., 2012).

Some other limitations of this study should also be noted. Comparison data was available for male 400 m runners only, not for female 400 m runners, and the sample size in the 1500 m races was small. In the comparison of data, we were unable to adjust for all potential confounders that may affect pacing and velocity, such as age and training history. These limitations, however, do not alter the importance of our findings, as this study was the first to show a clear difference in pacing strategy during high level running competition between athletes with and without II, in particular in the longer distances, in which pacing and self-regulation becomes more crucial. These findings have contributed to the development of sport specific classification systems and hence created opportunities for athletes with intellectual impairment the world over to participate at the highest level of competition, i.e., The Paralympic Games (Kwon and Block, 2012).

In conclusion, elite runners with intellectual impairments run at an overall slower velocity and following a significantly different pacing pattern compared to runners without an intellectual impairment. For the 400 m race, the initial acceleration and the final deceleration observed in World record and World Championships races (runners without intellectual impairment) are less pronounced in the finals of high level competitions for runners with intellectual impairment. During the 1500 m race, both group of runners exhibit a seemingly inverse pacing profile. Large inter and intra-individual variations and fluctuations in velocity have been observed in runners with intellectual impairment. Our findings support the assumption that runners with impaired cognitive abilities are less able to regulate their exercise intensity over the race than typical runners, even if they are equally well-trained.

## Author contributions

DV: Conceptualizing and drafting the article, revising it critically for important intellectual content, final approval of the version to be published, and accountability for all aspects of the work. FH, KM, and YV: Conceptualizing and revising the study critically for important intellectual content, final approval of the version to be published, and accountability for all aspects of the work.

## Funding

The first author is supported by the International Paralympic Committee (IPC). No other sources of funding were used to assist in the preparation of this study.

### Conflict of interest statement

The authors declare that the research was conducted in the absence of any commercial or financial relationships that could be construed as a potential conflict of interest.
